# Complete Mitogenomes of the Critically Endangered Hainan Gibbon (*Nomascus hainanus*) Reveal Very Low Mitochondrial Diversity and Two Major Maternal Clades

**DOI:** 10.3390/ani16193091

**Published:** 2026-10-01

**Authors:** Zhonghao Yu, Ren Xiahou, Meng Nie, Xiaoyi Cai, Dingfeng Duan, Yanjiao Zhou, Yuan Chen, Wenxing Long, Chenghong Liao

**Affiliations:** 1 Laboratory of Tropical Veterinary Medicine and Vector Biology, School of Life and Health Sciences, Hainan Province Key Laboratory of One Health, Collaborative Innovation Center of One Health, Hainan University, Haikou 570228, China; 15607532820@163.com (Z.Y.);; 2 Hainan International One Health Institute, Hainan University, Haikou 570228, China; 3 Wuzhishan National Long-Term Forest Ecosystem Monitoring Research Station, Hainan Key Laboratory for Sustainable Utilization of Tropical Bioresource, College of Forestry, Hainan University, Haikou 570228, China; 4 Institute of Hainan National Park, Haikou 570100, China

**Keywords:** *Nomascus hainanus*, mitochondrial genome, maternal clade, founder effect, divergence time, purifying selection

## Abstract

The Hainan gibbon (*Nomascus hainanus*) is one of the most threatened primates, yet its mitochondrial haplotype diversity and genealogy remain poorly understood. This study investigated mitochondrial haplotype structure, genealogical history, and selective constraints on mitochondrial protein-coding genes using complete mitochondrial genomes. We generated mitogenomic data from 13 wild individuals representing five of seven extant social groups, including the recently formed F and G groups, which were established through natural dispersal and had not previously been genetically characterized. Complete mitogenomes were obtained using a combination of Illumina and Sanger sequencing, followed by population genetic analyses, phylogenetic reconstruction, molecular dating, and mitochondrial selection tests. All mitogenomes were 16,481 bp in length and exhibited very low nucleotide diversity (π = 0.000498), with four haplotypes forming two major maternal clades. Groups B, C, and E contained haplotypes from both major maternal clades, whereas F and G each showed a single sampled haplotype, a pattern consistent with maternal founder effects in these recently formed groups. The two major maternal clades had an estimated time to the most recent common ancestor (TMRCA) of ~27 ka (95% highest posterior density (HPD): 11.9–45.7 ka), placing their most recent common ancestor in the Late Pleistocene. Interspecific selection analyses were consistent with predominantly purifying selection across mitochondrial protein-coding genes, with no significant evidence of episodic positive selection or altered selection intensity. These findings complement previous nuclear genomic and partial mitochondrial DNA studies by providing a population-level view of complete-mitogenome haplotype distribution across five sampled social groups.

## Simple Summary

The Hainan gibbon (*Nomascus hainanus)* is one of the world’s most endangered primates, with its population now restricted to a small remnant of its former range after long-term habitat loss and human disturbance. In this study, we generated and analyzed complete mitochondrial genomes from 13 wild Hainan gibbons representing five extant social groups. We identified two major maternal clades that have persisted despite severe population decline. Newly established social groups each contained a single sampled maternal haplotype, showing a pattern consistent with maternal founder effects during their establishment through natural dispersal. In addition, interspecific analyses were consistent with predominantly purifying selection across mitochondrial protein-coding genes. These findings provide new insights into the maternal evolutionary history of Hainan gibbons and characterize the maternal variation retained across extant social groups.

## 1. Introduction

The Hainan gibbon (*Nomascus hainanus*), an island-endemic primate unique to Hainan Island of China, is classified as Critically Endangered by the International Union for Conservation of Nature (IUCN) Red List and represents the world’s rarest ape species [1]. Massive population declines occurred throughout the 20th century driven by deforestation, habitat fragmentation, and persistent human disturbance, restricting the surviving population to a small remnant area within its former range [2]. A 2026 nuclear genomic study estimated the current wild population at approximately 42 individuals [3]. Although targeted conservation interventions have enabled slow population recovery over the past two decades, the population declined to only seven to eight individuals in the 1970s and remains highly vulnerable because of its small population size, restricted habitat, slow reproductive rate, very low genetic diversity, and potential risk of future inbreeding [4,5]. Microsatellite studies estimated contemporary effective population size (N_e_) values of 2.16 and 5.3 and detected substantial genetic diversity loss and evidence of recent and earlier bottlenecks [5,6]. Genetic analyses can help characterize the consequences of this decline, while habitat protection and restoration remain the primary conservation priorities [1,4,6].

**Figure 1:**
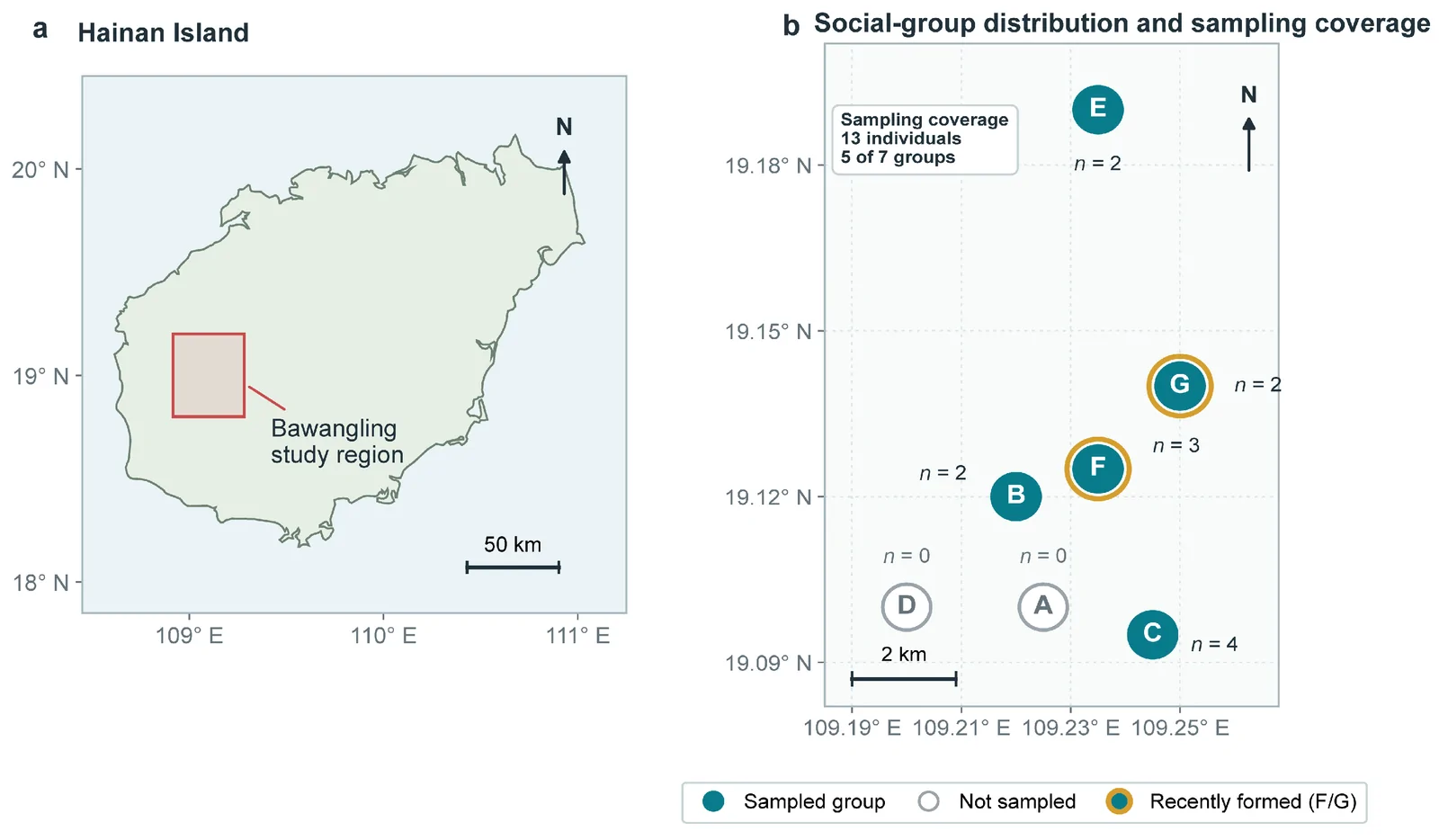
Study area, spatial distribution of extant Hainan gibbon social groups, and sampling coverage. (**a**) Location of the Bawangling study region on Hainan Island. (**b**) Approximate spatial distribution of the seven extant social groups (A–G) within the study region. Filled teal circles indicate groups sampled in this study (B, C, E, F, and G), open gray circles indicate unsampled groups (A and D), and gold outlines identify the recently formed Groups F and G. Values of *n* indicate the number of adult individuals sampled from each group.

Recent nuclear genomic and microsatellite research has clarified genome-wide population differentiation and recent gene flow patterns of *N. hainanus* [3,7]. Nuclear markers capture biparental inheritance and recombination [8,9]; nuclear genomic analyses identified two historically differentiated lineages, represented by Groups A and B, that diverged approximately 450 years ago [3]. In contrast, mitochondrial genomes are maternally inherited, providing a complementary perspective on maternal genealogical history, female-mediated dispersal, and founder effects during new group establishment [8,9]. Earlier short-fragment mitochondrial DNA (mtDNA) studies relying only on the control region preliminarily reported ultra-low genetic diversity and the existence of two mitochondrial haplotypes in this species [10]. However, population-level complete-mitogenome variation remains insufficiently characterized [3,7,10]. The current Bawangling population comprises seven extant social groups (A–G; Figure 1), among which the recently formed F and G groups were not represented in previous genetic studies [3,7,10]. Compared with short control-region fragments, complete mitogenomes provide broader sequence coverage for characterizing haplotypes, estimating their genealogy, and assessing selective constraints across mitochondrial protein-coding genes [8,9].

In this study, we generated complete mitochondrial genomes from 13 wild adult Hainan gibbons representing five of seven extant social groups, including the recently formed F and G groups. Our objectives were to characterize mitochondrial diversity and haplotypes, define the major maternal clades and estimate their time to the most recent common ancestor (TMRCA), infer mito-phylogenetic relationships, and characterize selective constraints on mitochondrial protein-coding genes (PCGs) through interspecific comparisons. This study provides a population-level view of complete-mitogenome haplotype variation across five sampled social groups and complements previous nuclear genomic and partial-mtDNA studies.

## 2. Materials and Methods

### 2.1. Sample Collection and Mitogenome Sequencing

Fecal samples were collected between December 2023 and June 2025. Thirteen adult wild Hainan gibbons from five of the seven extant social groups (B, C, E, F, and G; Figure 1) were sampled non-invasively (Table 1). According to continuous field-monitoring records maintained by the study team, the wild population comprised 42 individuals as of June 2025, including 21 adults across the seven social groups. All adults in Groups C, E, F, and G were sampled, whereas two of the four adults in Group B were included. Groups A and D were not sampled because of field-access constraints, including inaccessible terrain and limited individual encounter rates. Notably, groups F and G were recently established through natural dispersal and have not been included in previous population genetic studies. This study thus provides the first complete-mitogenome characterization of these newly established groups. Three-letter sample IDs indicate the social group, individual, and sex, respectively (F, female; M, male); for example, CBM denotes male individual B from Group C.

To reduce the risk of nuclear mitochondrial pseudogene (NUMT) contamination while retaining sequencing efficiency, we combined Illumina next-generation sequencing (NGS) and Sanger sequencing pipelines. Given the low proportion and degradation of host DNA in faecal samples, applying whole-genome shotgun sequencing to all individuals would have been less sequencing-efficient and substantially more costly. Of the 13 sampled individuals, C Group Female A (CAF) was selected as the reference individual for whole-genome shotgun sequencing on the Illumina NovaSeq 6000 platform (Illumina, Inc., San Diego, CA, USA). The mean sequencing depth across the assembled mitochondrial genome was 24.75×. Raw FASTQ reads were quality-filtered with fastp v0.20.1 [11], mitochondrial reads were de novo assembled with SPAdes v3.15 [12], and assembly contigs and circularization graphs were manually curated in Bandage v0.8.1 [13] to produce a closed circular reference mitogenome.

Species-specific Sanger amplification primers were designed against the reference genome (Table S1) to generate overlapping amplicons covering the complete mitogenomes of the remaining 12 individuals. All chromatograms were manually inspected to confirm base calls at polymorphic sites and screen for ambiguous or mixed signals potentially indicative of nonspecific amplification or NUMT co-amplification. Genome annotation was performed using PhyloSuite v1.2.2 [14], MITOS2 [15], and tRNAscan-SE v2.0 [16]; circular mitogenome visualization was generated via OGDRAW v1.3.1 [17].

### 2.2. Genetic Diversity, Haplotype Network, and Sliding Window Variation Analyses

Basic mitogenomic population statistics (nucleotide composition, segregating sites, nucleotide diversity π, haplotype diversity Hd, Tajima’s D) were calculated in DnaSP v6.12 [18]. A sliding window scan (window = 500 bp, step = 25 bp) was implemented to map regional variation of π across the 16,481 bp mitogenome. As a sensitivity analysis, the scan was repeated using a 1000-bp window with the same 25-bp step (Figure S2). Relative synonymous codon usage (RSCU) of the 13 mitochondrial PCGs was computed in MEGA X [19]. Median-joining haplotype networks were constructed in PopART v1.7 [20]. Single-nucleotide polymorphism (SNP) distribution heatmaps were visualized in R v4.2.1 (R Foundation for Statistical Computing, Vienna, Austria).

### 2.3. Selection Pressure Analyses

Mitochondrial PCG sequences of the comparative taxa were retrieved from NCBI GenBank and analyzed on the Datamonkey 2.0 platform [21]. Two complementary selection models were applied: (1) Branch-Site Unrestricted Statistical Test for Episodic Diversification (BUSTED) to detect gene-wide episodic diversifying positive selection [22]; (2) RELAX to test for significant relaxation or intensification of purifying selection, where K < 1 indicates relaxed constraints and K > 1 indicates strengthened selection [23]. RELAX was applied to the interspecific dataset, with branches belonging to *N. hainanus* designated as the test set and mainland *Nomascus* branches as the reference set. We additionally calculated gene-specific pairwise nonsynonymous-to-synonymous substitution-rate ratios (Ka/Ks; ω) between the *N. hainanus* reference mitogenome (PZ236455) and four *Nomascus* congeners (*N. concolor*, *N. gabriellae*, *N. leucogenys*, and *N. siki*) using the MYN and GY-HKY methods.

### 2.4. Phylogenetic Reconstruction and Divergence Time Estimation

Complete mitogenome sequences of the comparative taxa were retrieved from NCBI GenBank and aligned using MAFFT v7.487 [24] within PhyloSuite. Partitioning schemes and optimal substitution models were selected via ModelFinder [25]. Maximum Likelihood (ML) phylogenies were inferred in IQ-TREE v2.1.2 [26], with 1000 ultrafast bootstrap replicates for node support; Bayesian Inference (BI) trees were built in MrBayes v3.2.7 [27].

Divergence time estimation was performed in Bayesian Evolutionary Analysis Sampling Trees (BEAST) v1.10.4 [28]. Two secondary calibration points were applied: divergence between *N. hainanus* and the lineage containing *N. concolor* (mean = 2.14 million years ago (Mya), standard deviation (SD) = 0.22) and divergence between *N. concolor* and the lineage comprising *N. leucogenys*, *N. siki*, and *N. gabriellae* (mean = 1.68 Mya, SD = 0.17) [29]. These secondary calibration points were derived from previous molecular estimates, and their associated uncertainty was therefore propagated into the posterior divergence-time estimates. We adopted an uncorrelated lognormal relaxed molecular clock and Birth–Death tree prior [30,31]. Markov chain Monte Carlo (MCMC) chains ran for 100 million generations with sampling every 10,000 generations; convergence (effective sample size (ESS) > 200) was verified in Tracer v1.7 [32]. To address the wide 95% HPD interval of TMRCA, we conducted a sensitivity analysis by re-running BEAST with strict molecular clock and alternative outgroup sets to compare divergence time estimates. The final maximum clade credibility (MCC) tree was summarized in TreeAnnotator.

For comparative mitogenomic analyses, representative complete mitogenomes of four other *Nomascus* species (*N. concolor*, *N. leucogenys*, *N. siki*, and *N. gabriellae*) and representatives of *Hylobates*, *Hoolock*, and *Symphalangus* were retrieved from NCBI GenBank (Table S2). Genome length, A + T content, gene complement, and gene order were compared. Uncorrected p-distances were calculated from pairwise whole-mitogenome alignments, excluding positions containing gaps, and averaged across comparisons with the 13 *N. hainanus* mitogenomes.

### 2.5. Historical Demographic Analyses

Pairwise mismatch distribution analysis was performed in DnaSP v6 to characterize the distribution of pairwise sequence differences. Goodness-of-fit metrics (sum of squared deviations (SSD), Harpending’s raggedness index r) were calculated to assess compatibility with a simple demographic expansion model.

## 3. Results

### 3.1. Mitogenome Organization and Sequence Characteristics

**Figure 2:**
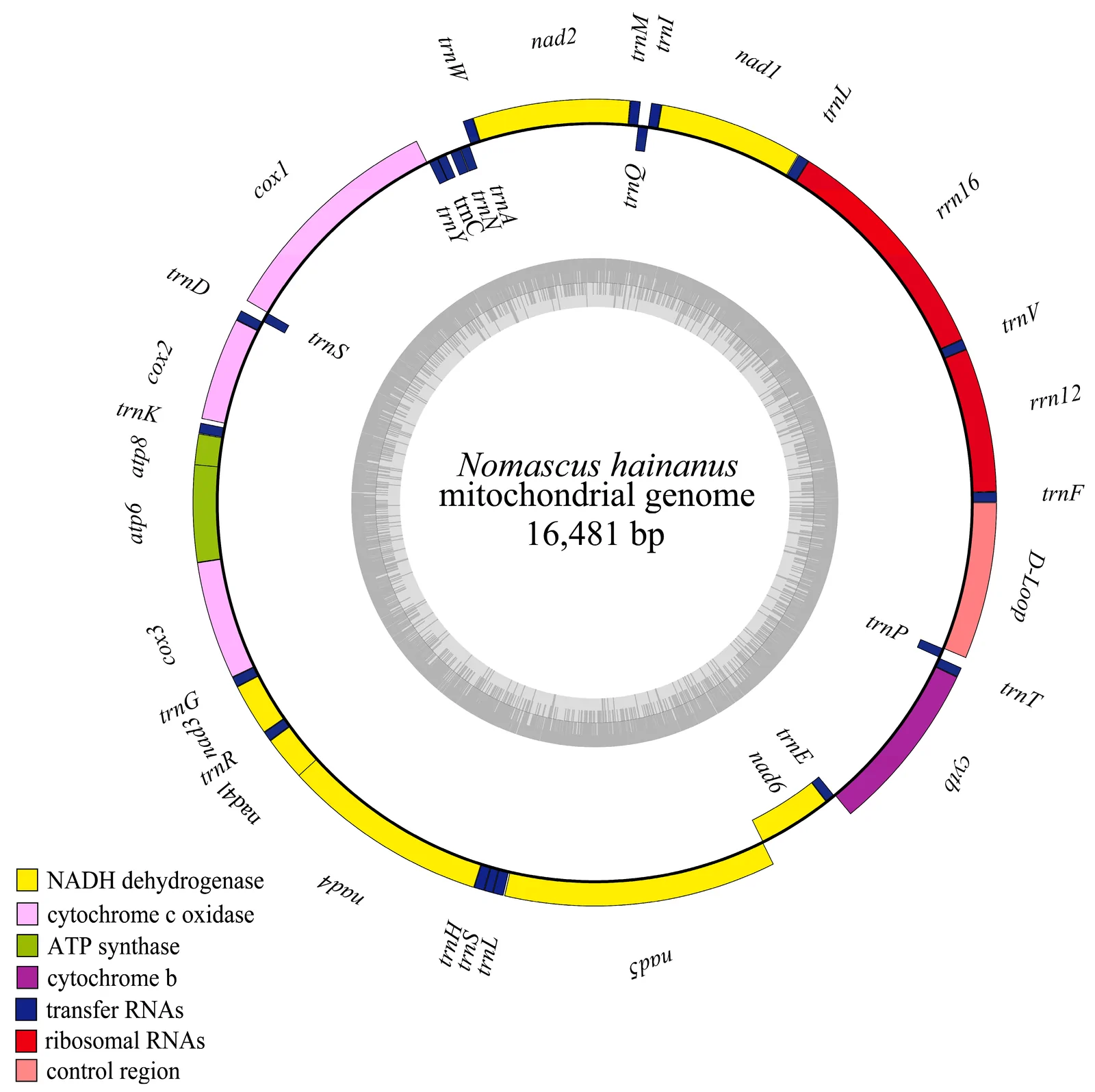
Complete mitochondrial genome map of the Hainan gibbon (*Nomascus hainanus*), spanning 16,481 bp. Genes depicted on the outer and inner rings are encoded on the heavy (H) and light (L) strands, respectively. Color-coded blocks denote functional categories—NADH dehydrogenase, cytochrome c oxidase, ATP synthase, rRNAs, and tRNAs—as indicated in the legend (lower left). The innermost grey ring represents the distribution of GC content.

All 13 sequenced mitogenomes were identical in total length (16,481 bp) with highly conserved structural architecture, containing the canonical 37 mitochondrial genes (13 PCGs, 22 tRNAs, 2 rRNAs, single control region). Across the 13 mitogenomes, mean nucleotide proportions were A = 30.69%, T = 24.08%, G = 13.72%, and C = 31.50%, with a mean A + T content of 54.77%; mean AT-skew and GC-skew values were 0.120 and −0.393, respectively. The circular mitogenome map is shown in Figure 2.

Direct comparison with representative Hylobatidae mitogenomes showed the same gene complement and gene order, with genome lengths of 16,468–16,516 bp and A + T contents of 54.55–56.48% (Table S2). The *N. hainanus* mitogenomes therefore fall within the structural and compositional range of the representative hylobatid mitogenomes examined. Mean complete-mitogenome p-distances between *N. hainanus* and the four other *Nomascus* species were 5.10–5.29%, whereas distances to representatives of *Symphalangus*, *Hoolock*, and *Hylobates* were 9.18–9.95%.

### 3.2. Very Low Mitogenomic Diversity and Two Major Maternal Clades

**Figure 3:**
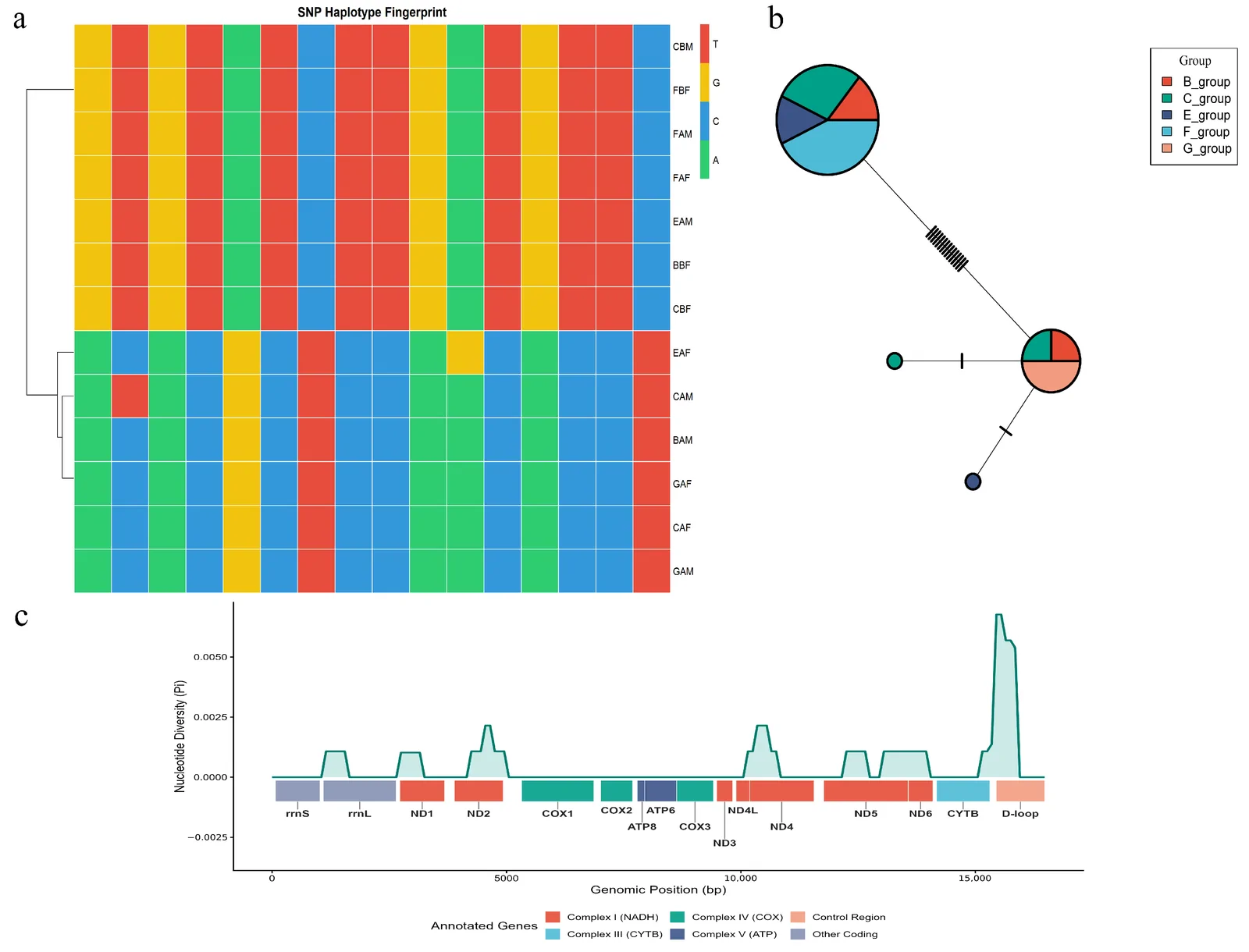
Maternal genetic polymorphism, major maternal clade structure, and nucleotide variation distribution in extant Hainan gibbons. (**a**) Single-nucleotide polymorphism (SNP) haplotype fingerprint heatmap. Rows represent 13 individuals grouped by social group (indicated on the right), and columns correspond to mitogenomic nucleotide polymorphisms, with bases color-coded. (**b**) Median-joining network of maternal haplotypes. Circle diameters are proportional to haplotype frequencies, and internal segments indicate the distribution of individuals across social groups (B, C, E, F, G). Tick marks along branches denote mutational steps. (**c**) Sliding window analysis of mitogenomic nucleotide diversity (π) using a 500-bp window with 25-bp steps. Gene boundaries corresponding to functional complexes are indicated along genomic coordinates. Sample labels (e.g., CBM) denote individual animals and are defined in Section 2.1.

Across the full 16,481-bp alignment, 16 biallelic SNPs were identified, defining four haplotypes (h = 4; Hd = 0.6538), with very low nucleotide diversity (π = 0.000498) and a significantly positive Tajima’s D (D = 2.4847, *p* = 0.013). Despite limited mitogenome-wide variation, phylogenetic trees and median-joining haplotype networks consistently grouped the four haplotypes into two major maternal clades (Figure 3a,b). Maternal haplotype composition differed among social groups: groups B, C, and E contained individuals from both major maternal clades, whereas the recently formed F and G groups each contained a single sampled maternal haplotype.

Sliding-window nucleotide diversity scans revealed uneven variation across the mitogenome. All cytochrome c oxidase and ATP synthase genes were invariant, whereas the non-coding control region showed the highest nucleotide diversity; additional polymorphic sites occurred in 16S rRNA and in *ND1*, *ND2*, *ND4*, *ND5*, *ND6*, and *CYTB* (Figure 3c). The 1000-bp sensitivity scan smoothed short-scale fluctuations but retained the same broad regional pattern, with the control region remaining the most variable region (Figure S2). Of the 16 variable sites, nine occurred within mitochondrial PCGs, of which seven were synonymous and two were nonsynonymous, corresponding to amino-acid differences in *ND4* (Tyr/Cys at residue 52) and *CYTB* (Ala/Thr at residue 380). Neither nonsynonymous variant was private to the recently formed F or G groups.

### 3.3. Conserved Codon Usage and Predominantly Purifying Selection

RSCU analysis showed nearly identical synonymous codon preference across all individuals and social groups. Synonymous codons terminating in A/C were preferentially utilized, while G/U-ending synonymous codons were rare (Figure S5). No apparent differences in codon bias were observed between the two major maternal clades or among social groups.

Interspecific selection analyses were consistent with predominantly purifying selection across mitochondrial PCGs. The BUSTED model returned non-significant results for episodic positive selection (*p* = 0.50), with no gene-wide diversifying selection signals. RELAX did not detect a statistically significant difference in selection intensity between *N. hainanus* and mainland *Nomascus* congeners (K = 1.030, *p* = 0.931). Gene-specific ω ratios from the interspecific comparisons between *N. hainanus* and four *Nomascus* congeners were consistently below 1 (mean ω = 0.049 under MYN and 0.062 under GY-HKY), providing descriptive support for predominantly purifying selection across mitochondrial PCGs.

### 3.4. Divergence Time Estimation and Mismatch Distribution

**Figure 4:**
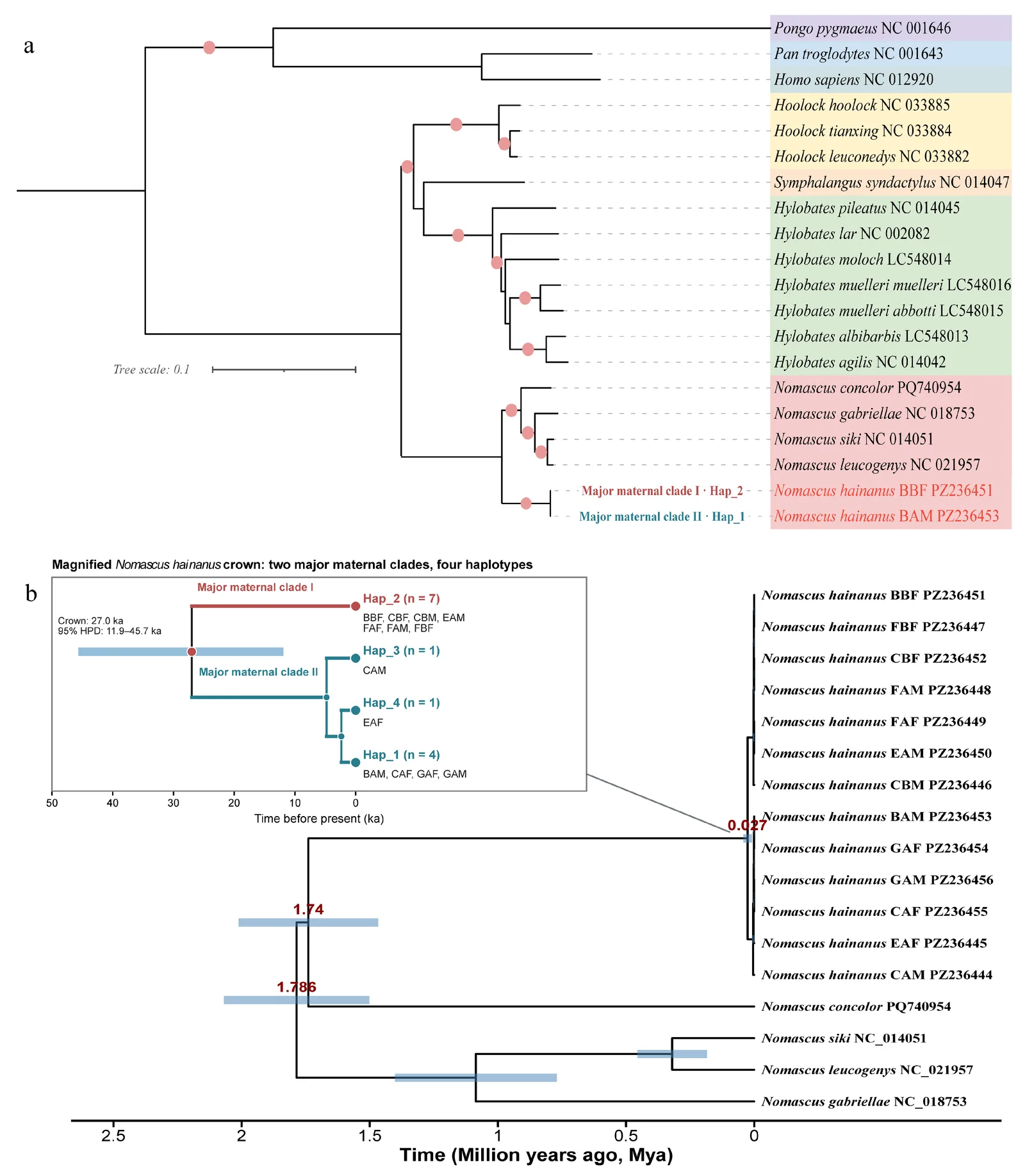
Mito-phylogenetic relationships and divergence time estimation of *Nomascus hainanus* based on complete mitogenomes. (**a**) Maximum-likelihood (ML) tree inferred using IQ-TREE, corroborated by congruent Bayesian inference (BI) topology. Pink circles indicate nodes with high support; colored blocks delineate taxonomic clades. (**b**) BEAST-derived Maximum Clade Credibility (MCC) tree for *Nomascus* species and the four mitochondrial haplotypes of *N. hainanus*. Red numbers denote time-to-the-most-recent-common-ancestor (TMRCA) estimates in million years ago (Mya), and blue bars represent 95% highest posterior density (HPD) intervals. Solid lines indicate phylogenetic branches, whereas gray dashed lines connect terminal branches to taxon labels.

The ML phylogeny based on complete mitogenomes placed the two representative *N. hainanus* haplotypes within the *Nomascus* clade, with a topology congruent with the BI analysis (Figure 4a and Figure S4). BEAST dating estimated the TMRCA of the two major maternal clades at ~27 ka (95% HPD: 11.9–45.7 ka), placing their most recent common ancestor in the Late Pleistocene. Sensitivity analyses using a strict molecular clock and alternative outgroup sets yielded TMRCA estimates of 22–31 ka, broadly consistent with the main estimate of ~27 ka (Figure 4b).

Mismatch distribution analysis showed a bimodal pattern, with peaks at 0–1 and 14–16 pairwise differences, corresponding mainly to within- and between-clade comparisons, respectively (Figure S6). The distribution did not show the simple unimodal pattern expected under a recent sudden-expansion model; the SSD was 0.215, and Harpending’s raggedness index was 0.2286.

## 4. Discussion

### 4.1. Temporal Depth and Distribution of the Two Major Maternal Clades

Comprehensive analyses of complete mitochondrial genomes demonstrate that extant Hainan gibbons retain two major maternal clades despite the recent population decline. These clades occur against a background of very low population-level mitochondrial diversity but preserve maternal genealogical structure that predates the recent population decline. The estimated TMRCA of the two maternal clades, approximately 27 ka, places their shared maternal ancestry in the Late Pleistocene but does not imply extensive sequence divergence. Recent nuclear genomic analyses identified a prolonged population bottleneck associated with the Last Glacial Maximum, followed by a millennial-scale population expansion [3]. This demographic history provides a plausible context for the persistence of the two maternal clades [3]. However, temporal correspondence alone is insufficient to attribute their divergence to a specific climatic or demographic event.

Across the 16,481-bp mitogenome, the two major maternal clades differed by 14–16 substitutions, corresponding to a mean uncorrected p-distance of 0.091% (range: 0.085–0.097%). In contrast, mean p-distances between *N. hainanus* and the four other *Nomascus* species were 5.10–5.29% (Table S2). Thus, the two maternal clades are distinguishable within the species but show limited sequence differentiation. The ~27-ka TMRCA therefore reflects the temporal depth of the surviving maternal genealogy rather than extensive sequence divergence.

At the population level, nucleotide diversity in *N. hainanus* (π = 0.000498) was lower than published whole-mitogenome values for *Hylobates* (π = 0.00071–0.01671) and sampled *Nomascus* taxa (π = 0.00253–0.00432), including *N. concolor* (π = 0.00253) [29]. Although differences in sampling design and sequence processing limit direct comparability among studies, these values provide a broader hylobatid context for the very low mitochondrial diversity observed in *N. hainanus* [29].

Census size, historical minimum population size, and effective population size (N_e_) describe different aspects of population status [33]. The mitogenomic data presented here characterize the maternal variation retained in the current population and do not independently estimate bottleneck duration. Behavioral observations have documented variation in group composition and mating strategies in *N. hainanus* [34], but whether these patterns generate population-level reproductive skew and further reduce N_e_ remains unknown because population-wide reproductive-success data are unavailable [33].

These results complement, rather than supplant, recent nuclear genomic studies of the species. Hou et al. [3] inferred an approximately 450-year split between nuclear genomic lineages, whereas our ~27-ka mitochondrial estimate represents the TMRCA of the two major maternal clades. Because mitochondrial coalescence can occur before population divergence owing to the persistence of ancestral polymorphism [9], these estimates are not directly comparable. The ~27-ka estimate therefore reflects the temporal depth of the surviving maternal genealogy rather than the timing of population separation.

The bimodal mismatch distribution and significantly positive Tajima’s D were inconsistent with a simple recent population-expansion model [35,36]. Given the limited number of variable sites and the sensitivity of both statistics to population structure [35,36], the specific demographic processes underlying these patterns remain unresolved.

### 4.2. Co-Occurrence of Major Maternal Clades Among Social Groups and Maternal Founder-Effect Patterns in Recently Formed F/G Groups

Patterns of mitochondrial haplotype distribution among social groups offer further insight into recent population dynamics. Groups B, C, and E contained individuals from both major maternal clades. This pattern may reflect retained ancestral maternal variation, subsequent dispersal and group formation, or both [3,34]. Nuclear genomic analyses identified two historically differentiated lineages represented by Groups A and B, which diverged approximately 450 years ago with no detectable gene flow before the formation of Group C [3]. Pedigree reconstruction further showed that Group C was founded by emigrants from Groups A and B, whereas Group E was subsequently established by individuals originating from Groups C and B [3]. These findings support recent dispersal and group formation as an explanation for the co-occurrence of the two maternal clades in Groups C and E [3]. For Group B, however, mitochondrial data alone cannot distinguish retained ancestral variation from later immigration. Available behavioral evidence indicates that group formation and membership can involve dispersal, social reorganization, and adult immigration or replacement rather than simple fission of intact social groups [5,34]. Thus, social-group boundaries do not necessarily imply permanent demographic isolation [3,34]. However, the available data are insufficient to predict future patterns of group formation and isolation. These uncertainties highlight the importance of maintaining habitat connectivity and continuing to monitor dispersal and group formation during population recovery [1,4].

In contrast, the recently formed F and G groups each contained a single sampled maternal haplotype within each group, a pattern consistent with maternal founder effects. Because mtDNA is maternally inherited, this pattern reflects only the maternal component of recent group establishment and does not resolve the full dispersal history or male-mediated movement [8,9].

### 4.3. Selective Constraints on Mitochondrial Protein-Coding Genes in the Context of Small Population Size

Small bottlenecked populations are theoretically predicted to suffer weakened purifying selection, allowing mildly deleterious mitochondrial mutations to accumulate via genetic drift [37]. Complete-mitogenome studies of other severely bottlenecked taxa have documented substantial reductions in mitochondrial diversity [38,39]. Mountain gorillas exhibited very low mitochondrial diversity in both historical and modern samples, with four historical haplotypes but only two modern haplotypes [38]. Kākāpō also experienced substantial loss of mitochondrial diversity following recent anthropogenic population decline, although no evidence for fixation of deleterious mutations in mitochondrial coding regions was detected [39]. Differences in temporal sampling and analytical design preclude direct quantitative comparison with *N. hainanus* [38,39], but these studies provide a relevant mitochondrial context for interpreting the consequences of severe population decline.

At the interspecific level, RELAX did not detect a statistically significant difference in selection intensity between *N. hainanus* and mainland *Nomascus* congeners (K = 1.030, *p* = 0.931), while Ka/Ks estimates were consistently below 1, providing descriptive support for predominantly purifying selection across mitochondrial PCGs. The two nonsynonymous differences identified in *ND4* and *CYTB* were shared with maternal haplotypes represented in established groups and were not private to the recently formed F or G groups. Therefore, no F/G-specific amino-acid change or group-specific functional effect on the respiratory chain could be inferred. Overall, the interspecific results support the evolutionary conservation of mitochondrial PCGs, whereas limited intraspecific variation prevents firm conclusions about recent changes in selection intensity.

### 4.4. Scope and Limitations of Mitogenomic Inference

The present study provides a population-level view of maternal variation in the Hainan gibbon. The distribution of the two major maternal clades among social groups shows how surviving maternal variation is structured within the current population, while the presence of a single sampled maternal haplotype within each of the recently formed F and G groups is consistent with maternal founder effects during recent group formation. When combined with nuclear genomic and field data, these findings provide maternal genetic evidence relevant to conservation-unit assessment and genetic-risk evaluation [4,6], while habitat protection, restoration, and expansion remain the primary conservation priorities [1,4].

Nevertheless, these results should be interpreted within the inherent limitations of mitochondrial data. As mitochondrial genomes reflect strictly maternal inheritance, they cannot provide a comprehensive assessment of genome-wide diversity, levels of inbreeding, adaptive potential, or the demographic consequences of male-mediated gene flow [8,9]. Divergence time estimates are similarly dependent on secondary calibrations and should therefore be considered approximate rather than definitive [40]. Integrating nuclear genomic analyses, pedigree data, and ecological monitoring will be essential for generating a more complete understanding of long-term population viability in this critically endangered primate [3,4,6].

NUMTs have been documented in primate nuclear genomes, although their prevalence in *Nomascus* has not been systematically evaluated [41]. Long-read sequencing could provide complementary validation; however, fecal DNA typically contains a low proportion of fragmented host DNA, abundant microbial and dietary DNA, and potential PCR inhibitors [42]. Consequently, recovering host DNA of sufficient molecular weight, purity, and quantity for long-read sequencing can be challenging [42,43].

Overall, mitogenome analyses show that the Hainan gibbon retains two major maternal clades, while interspecific analyses are consistent with predominantly purifying selection across mitochondrial PCGs. The distribution of maternal haplotypes among social groups also reflects patterns associated with recent dispersal and group formation.

## 5. Conclusions

Full-length mitochondrial genome sequencing of 13 wild Hainan gibbons revealed very low nucleotide diversity and four haplotypes forming two major maternal clades across five sampled social groups. The two clades had an estimated TMRCA of ~27 ka, placing their shared maternal ancestry in the Late Pleistocene. The recently formed F and G groups each contained a single sampled maternal haplotype, a pattern consistent with maternal founder effects during group establishment. Interspecific analyses were consistent with predominantly purifying selection across mitochondrial PCGs, but limited intraspecific variation prevented firm conclusions about recent changes in selection intensity.

This mitogenome dataset complements recent nuclear genomic research by providing a dedicated maternal evolutionary perspective on surviving haplotypes, maternal clade history, and recent group formation. When integrated with nuclear genomic and field data, these findings can contribute to broader genetic assessments, while habitat protection, restoration, and expansion remain the primary conservation priorities. This study is constrained by incomplete sampling across the seven extant social groups, with Groups A and D not represented, and by the exclusive use of maternally inherited mitochondrial markers; future work incorporating museum ancient DNA and nuclear markers will further refine demographic and adaptive evolutionary inferences for this Critically Endangered primate.

## Figures and Tables

**Table 1:** Sample list for the 13 Hainan gibbon individuals analyzed in this study. The GenBank accession numbers correspond to complete mitogenomes generated in this study.

Sample ID	GenBank Accession No.	Social Group	Sex
CAM	PZ236444	C	Male
EAF	PZ236445	E	Female
CBM	PZ236446	C	Male
FBF	PZ236447	F	Female
FAM	PZ236448	F	Male
FAF	PZ236449	F	Female
EAM	PZ236450	E	Male
BBF	PZ236451	B	Female
CBF	PZ236452	C	Female
BAM	PZ236453	B	Male
GAF	PZ236454	G	Female
CAF	PZ236455	C	Female
GAM	PZ236456	G	Male

## Data Availability

The complete mitochondrial genome sequences generated in this study for the 13 Hainan gibbon individuals have been deposited in GenBank under accession numbers PZ236444–PZ236456. The raw Illumina whole-genome sequencing data generated for the reference individual C Group Female A (CAF) have been deposited in the NCBI Sequence Read Archive (SRA) under accession number SRR39852636, which is associated with BioProject accession number PRJNA1499724 and BioSample accession number SAMN61991868. The original Sanger sequencing chromatogram files generated for the remaining 12 individuals are available from Zenodo (DOI: 10.5281/zenodo.21622489). All other data supporting the findings of this study are available within the article and its Supplementary Materials.

## Supplementary Materials

The following supporting information can be downloaded at: https://www.mdpi.com/article/10.3390/ani16193091/s1, Figure S1: Illumina sequencing depth and coverage distribution; Figure S2: Sensitivity analysis of sliding-window nucleotide diversity; Figure S3: Secondary structures of mitochondrial tRNAs; Figure S4: Bayesian inference (BI) phylogenetic tree; Figure S5: Relative synonymous codon usage (RSCU); Figure S6: Mismatch distribution analysis; Table S1: Primer sequences; Table S2: Comparative mitogenomic characteristics of representative Hylobatidae taxa; Table S3: Annotation of the mitochondrial genome; Table S4: Codon usage bias.

## References

[1] Liu G., Lu X., Liu Z., Xie Z., Qi X., Zhou J., Hong X., Mo Y., Chan B.P.L., Chapman C.A. (2022). The critically endangered Hainan gibbon (*Nomascus hainanus*) population increases but not at the maximum possible rate. Int. J. Primatol..

[2] Zhou J., Wei F., Li M., Zhang J., Wang D., Pan R. (2005). Hainan black-crested gibbon is headed for extinction. Int. J. Primatol..

[3] Hou X., Pan S., Xu J.L., Hu L., He W., Liu X., Huang S., Gu Z., Lin Z., Chen Y. (2026). Genetic legacy and recent cross of two ancient lineages underlie the rebound of the world’s rarest primate. Sci. Adv..

[4] Zhong X., Huang X., Zhu C., Wang Y., Chapman C.A., Garber P.A., Chen Y., Fan P. (2025). Science-based suggestions to save the world’s rarest primate species *Nomascus hainanus*. Sci. Adv..

[5] Guo Y., Chang J., Han L., Liu T., Li G., Garber P.A., Xiao N., Zhou J. (2020). The genetic status of the critically endangered Hainan gibbon (*Nomascus hainanus*): A species moving toward extinction. Front. Genet..

[6] Bryant J.V., Gottelli D., Zeng X., Hong X., Chan B.P.L., Fellowes J.R., Zhang Y., Luo J., Durrant C., Geissmann T. (2016). Assessing current genetic status of the Hainan gibbon using historical and demographic baselines: Implications for conservation management of species of extreme rarity. Mol. Ecol..

[7] Li W., Deng H., Zhou J. (2026). Genetic diversity of the critically endangered *Nomascus hainanus* based on non-invasive sampling microsatellite analysis. Am. J. Primatol..

[8] Galtier N., Nabholz B., Glémin S., Hurst G.D.D. (2009). Mitochondrial DNA as a marker of molecular diversity: A reappraisal. Mol. Ecol..

[9] Moritz C., Dowling T.E., Brown W.M. (1987). Evolution of animal mitochondrial DNA: Relevance for population biology and systematics. Annu. Rev. Ecol. Syst..

[10] Guo Y., Peng D., Han L., Liu T., Li G., Garber P.A., Zhou J. (2021). Mitochondrial DNA control region sequencing of the critically endangered Hainan gibbon (*Nomascus hainanus*) reveals two female origins and extremely low genetic diversity. Mitochondrial DNA Part B Resour..

[11] Chen S., Zhou Y., Chen Y., Gu J. (2018). fastp: An ultra-fast all-in-one FASTQ preprocessor. Bioinformatics.

[12] Bankevich A., Nurk S., Antipov D., Gurevich A.A., Dvorkin M., Kulikov A.S., Lesin V.M., Nikolenko S.I., Pham S., Prjibelski A.D. (2012). SPAdes: A new genome assembly algorithm and its applications to single-cell sequencing. J. Comput. Biol..

[13] Wick R.R., Schultz M.B., Zobel J., Holt K.E. (2015). Bandage: Interactive visualization of de novo genome assemblies. Bioinformatics.

[14] Zhang D., Gao F., Jakovlić I., Zou H., Zhang J., Li W.X., Wang G.T. (2020). PhyloSuite: An integrated and scalable desktop platform for streamlined molecular sequence data management and evolutionary phylogenetics studies. Mol. Ecol. Resour..

[15] Bernt M., Donath A., Jühling F., Externbrink F., Florentz C., Fritzsch G., Pütz J., Middendorf M., Stadler P.F. (2013). MITOS: Improved de novo metazoan mitochondrial genome annotation. Mol. Phylogenetics Evol..

[16] Chan P.P., Lowe T.M. (2019). tRNAscan-SE: Searching for tRNA genes in genomic sequences. Methods Mol. Biol..

[17] Greiner S., Lehwark P., Bock R. (2019). OrganellarGenomeDRAW (OGDRAW) version 1.3.1: Expanded toolkit for the graphical visualization of organellar genomes. Nucleic Acids Res..

[18] Rozas J., Ferrer-Mata A., Sánchez-DelBarrio J.C., Guirao-Rico S., Librado P., Ramos-Onsins S.E., Sánchez-Gracia A. (2017). DnaSP 6: DNA sequence polymorphism analysis of large data sets. Mol. Biol. Evol..

[19] Kumar S., Stecher G., Li M., Knyaz C., Tamura K. (2018). MEGA X: Molecular evolutionary genetics analysis across computing platforms. Mol. Biol. Evol..

[20] Leigh J.W., Bryant D. (2015). Popart: Full-feature software for haplotype network construction. Methods Ecol. Evol..

[21] Weaver S., Shank S.D., Spielman S.J., Li M., Muse S.V., Kosakovsky Pond S.L. (2018). Datamonkey 2.0: A modern web application for characterizing selective and other evolutionary processes. Mol. Biol. Evol..

[22] Murrell B., Weaver S., Smith M.D., Wertheim J.O., Murrell S., Aylward A., Eren K., Pollner T., Martin D.P., Smith D.M. (2015). Gene-wide identification of episodic selection. Mol. Biol. Evol..

[23] Wertheim J.O., Murrell B., Smith M.D., Pond S.L.K., Scheffler K. (2015). RELAX: Detecting relaxed selection in a phylogenetic framework. Mol. Biol. Evol..

[24] Katoh K., Misawa K., Kuma K.-I., Miyata T. (2002). MAFFT: A novel method for rapid multiple sequence alignment based on fast Fourier transform. Nucleic Acids Res..

[25] Kalyaanamoorthy S., Minh B.Q., Wong T.K.F., Von Haeseler A., Jermiin L.S. (2017). ModelFinder: Fast model selection for accurate phylogenetic estimates. Nat. Methods.

[26] Nguyen L.T., Schmidt H.A., von Haeseler A., Minh B.Q. (2015). IQ-TREE: A fast and effective stochastic algorithm for estimating maximum-likelihood phylogenies. Mol. Biol. Evol..

[27] Ronquist F., Teslenko M., van der Mark P., Ayres D.L., Darling A., Höhna S., Larget B., Liu L., Suchard M.A., Huelsenbeck J.P. (2012). MrBayes 3.2: Efficient bayesian phylogenetic inference and model choice across a large model space. Syst. Biol..

[28] Drummond A.J., Rambaut A. (2007). BEAST: Bayesian evolutionary analysis by sampling trees. BMC Evol. Biol..

[29] Liu S., Li K., Zheng Y., Xue J., Wang S., Li S., Cao P., Liu F., Dai Q., Feng X. (2025). Mitogenomes of museum specimens provide new insight into species classification and recently reduced diversity of highly endangered *Nomascus* gibbons. Integr. Zool..

[30] Drummond A.J., Ho S.Y.W., Phillips M.J., Rambaut A. (2006). Relaxed phylogenetics and dating with confidence. PLoS Biol..

[31] Heled J., Drummond A.J. (2015). Calibrated birth–death phylogenetic time-tree priors for bayesian inference. Syst. Biol..

[32] Rambaut A., Drummond A.J., Xie D., Baele G., Suchard M.A. (2018). Posterior summarization in bayesian phylogenetics using Tracer 1.7. Syst. Biol..

[33] Frankham R. (1995). Effective population size/adult population size ratios in wildlife: A review. Genet. Res..

[34] Li P., Garber P.A., Bi Y., Jin K., Qi X., Zhou J. (2022). Diverse grouping and mating strategies in the Critically Endangered Hainan gibbon (*Nomascus hainanus*). Primates.

[35] Simonsen K.L., Churchill G.A., Aquadro C.F. (1995). Properties of statistical tests of neutrality for DNA polymorphism data. Genetics.

[36] Excoffier L. (2004). Patterns of DNA sequence diversity and genetic structure after a range expansion: Lessons from the infinite-island model. Mol. Ecol..

[37] Popadin K., Polishchuk L.V., Mamirova L., Knorre D., Gunbin K. (2007). Accumulation of slightly deleterious mutations in mitochondrial protein-coding genes of large versus small mammals. Proc. Natl. Acad. Sci. USA.

[38] van der Valk T., Sandoval-Castellanos E., Caillaud D., Ngobobo U., Binyinyi E., Nishuli R., Stoinski T., Gilissen E., Sonet G., Semal P. (2018). Significant loss of mitochondrial diversity within the last century due to extinction of peripheral populations in eastern gorillas. Sci. Rep..

[39] Dussex N., von Seth J., Robertson B.C., Dalén L. (2018). Full Mitogenomes in the Critically Endangered Kākāpō Reveal Major Post-Glacial and Anthropogenic Effects on Neutral Genetic Diversity. Genes.

[40] Schenk J.J. (2016). Consequences of Secondary Calibrations on Divergence Time Estimates. PLoS ONE.

[41] Dayama G., Zhou W., Prado-Martinez J., Marques-Bonet T., Mills R.E. (2020). Characterization of nuclear mitochondrial insertions in the whole genomes of primates. NAR Genom. Bioinform..

[42] Chiou K.L., Bergey C.M. (2018). Methylation-based enrichment facilitates low-cost, noninvasive genomic scale sequencing of populations from feces. Sci. Rep..

[43] Wanner N., Larsen P.A., McLain A., Faulk C. (2021). The mitochondrial genome and epigenome of the golden lion tamarin from fecal DNA using Nanopore adaptive sequencing. BMC Genom..

