# Supplementary material for: Complete Mitogenomes of the Critically Endangered Hainan Gibbon (Nomascus hainanus) Reveal Very Low Mitochondrial Diversity and Two Major Maternal Clades
Source: Animals (Basel). 2026 Oct 1;16(19):3091. doi: 10.3390/ani16193091 (PMC13663578; doi:10.3390/ani16193091)
Supplement: Supplementary file 1 [file animals-16-03091-s001.zip › animals-4498649-supplementary.pdf]

Article

Complete Mitogenomes of the Critically Endangered Hainan Gibbon (*Nomascus hainanus*) Reveal Very Low Mitochondrial Diversity and Two Major Maternal Clades

Zhonghao Yu [1,2], Ren Xiahou[1,2], Meng Nie [1,2], Xiaoyi Cai [1,2], Dingfeng Duan [1,2], Yanjiao Zhou [1,2], Yuan Chen[3,4], Wenxing Long[3,4] and Chenghong Liao [1,2,*]

1 Laboratory of Tropical Veterinary Medicine and Vector Biology, School of Life and Health Sciences, Hainan Province Key Laboratory of One Health, Collaborative Innovation Center of One Health, Hainan University, Haikou, China
2 Hainan International One Health Institute, Hainan University, Haikou, China
3 Wuzhishan National Long-Term Forest Ecosystem Monitoring Research Station, Hainan Key Laboratory for Sustainable Utilization of Tropical Bioresource, College of Forestry, Hainan University, Haikou, China.
4 Institute of Hainan National Park, Haikou, China.
* Correspondence: liaochh@hainanu.edu.cn;

Keywords: *Nomascus hainanus*; mitochondrial genome; maternal clade; founder effect; divergence time; purifying selection

Number of Figures: 6 (S1, S2, S3, S4, S5, S6)
Number of Tables: 4 (S1, S2, S3, S4)

Academic Editor: Firstname Lastname

Received: date
Revised: date
Accepted: date
Published: date

Citation: To be added by editorial staff during production.

Copyright: © 2026 by the authors. Submitted for possible open access publication under the terms and conditions of the Creative Commons Attribution (CC BY) license (https://creativecommons.org/licenses/by/4.0/).

Figure S1. Illumina sequencing depth plot

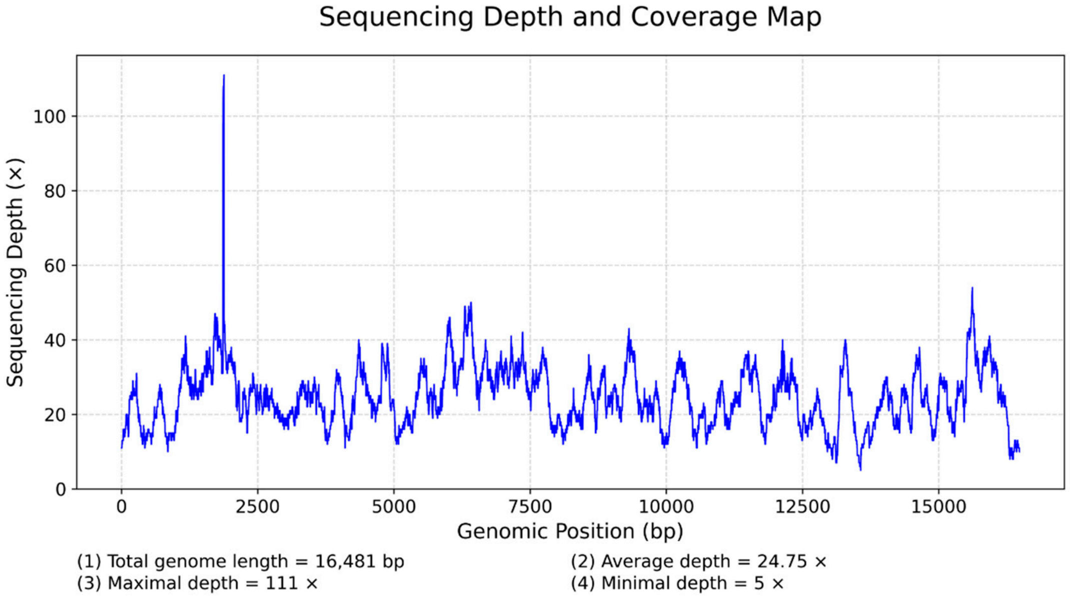

Figure S1. Illumina sequencing depth and coverage distribution of the Hainan gibbon (Nomascus hainanus) mitochondrial genome. The sequencing depth was calculated across the complete mitochondrial genome (16,481 bp) of the reference individual CAF. The average sequencing depth was 24.75×, with a maximum depth of 111× and a minimum depth of 5×.

Figure S2. Sensitivity analysis of sliding-window nucleotide diversity

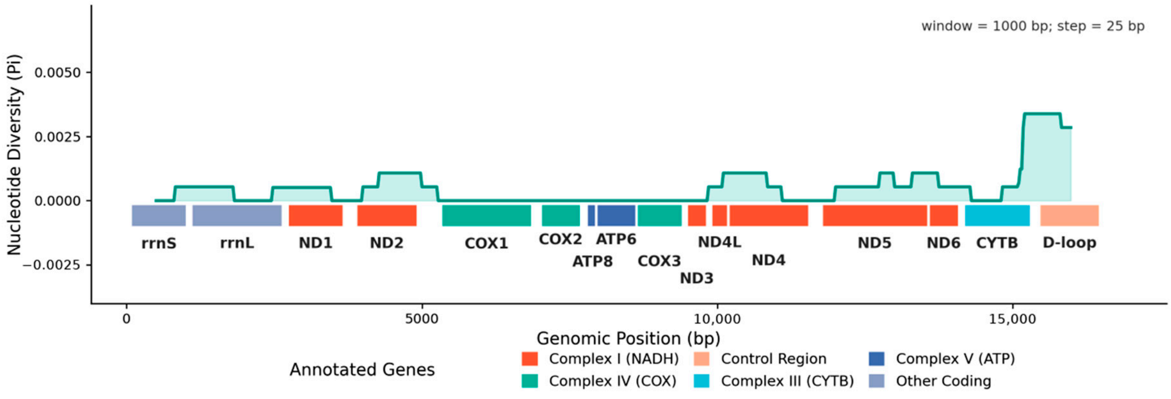

Figure S2. Sensitivity analysis of sliding-window nucleotide diversity across the Hainan gibbon (Nomascus hainanus) mitogenome using a 1000-bp window and 25-bp step. Relative to the primary 500-bp scan, the larger window smooths short-scale fluctuations while retaining the broad regional pattern, with the control region remaining the most variable region.

Figure S3. Secondary structure of Hainan gibbon tRNA

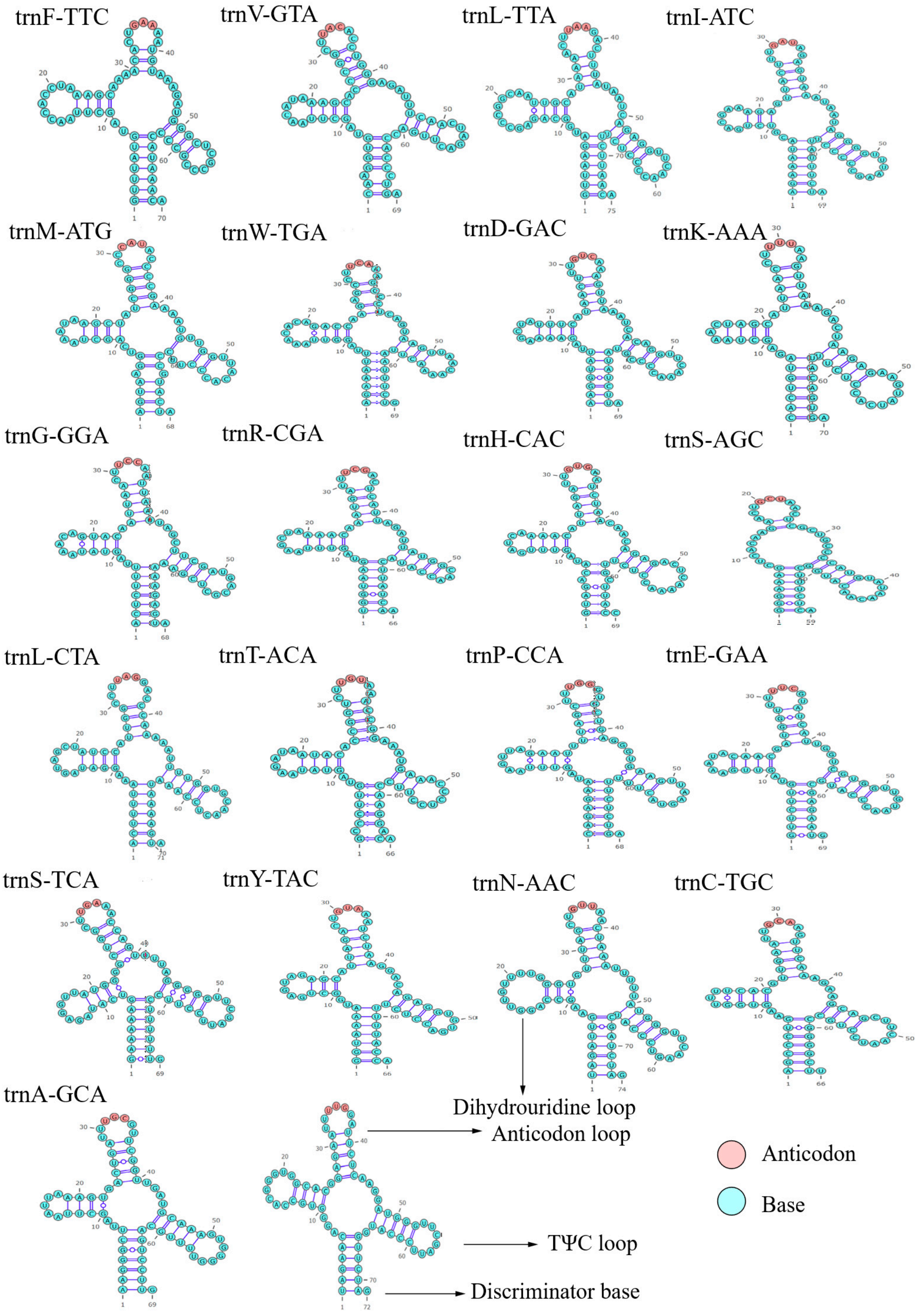

Figure S3. Secondary structures of mitochondrial tRNAs of the Hainan gibbon (*Nomascus hainanus*). The predicted secondary structures of 22 mitochondrial tRNAs are shown. Nucleotide positions and conserved structural features are indicated according to the standard cloverleaf model of tRNA. Different colors represent different nucleotide pairing states and structural features.

Figure S4. Bayesian inference (BI) tree constructed using MrBayes

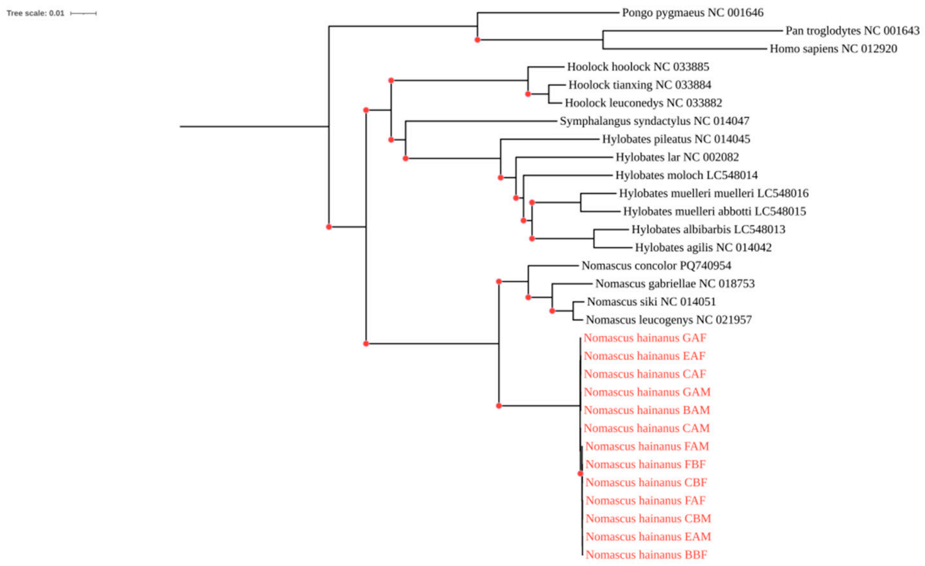

Figure S4. Bayesian inference (BI) phylogenetic tree based on complete mitochondrial genome sequences. The tree was constructed using MrBayes, and Bayesian posterior probabilities are shown at the nodes. Hainan gibbon (*Nomascus hainanus*) samples analyzed in this study are highlighted in red. Nodes with posterior probabilities ≥0.95 were considered strongly supported.

Figure S5. Relative synonymous codon usage (RSCU)

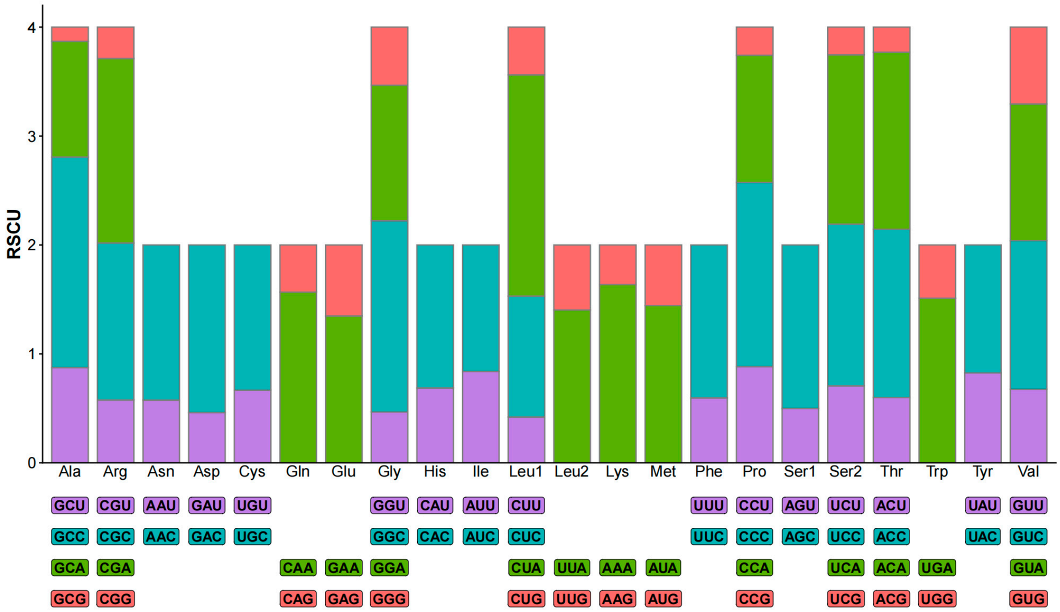

Figure S5. Relative synonymous codon usage (RSCU) in mitochondrial protein-coding genes of the Hainan gibbon (Nomascus hainanus). The x-axis represents amino acids, while the y-axis indicates corresponding RSCU values. Stacked bars depict the distribution of RSCU across synonymous codons, with distinct colors representing third-position (wobble) nucleotides: purple (U), cyan (C), green (A), and pink (G). Individual codons are labeled along the x-axis.

Figure S6. Mismatch distribution plot

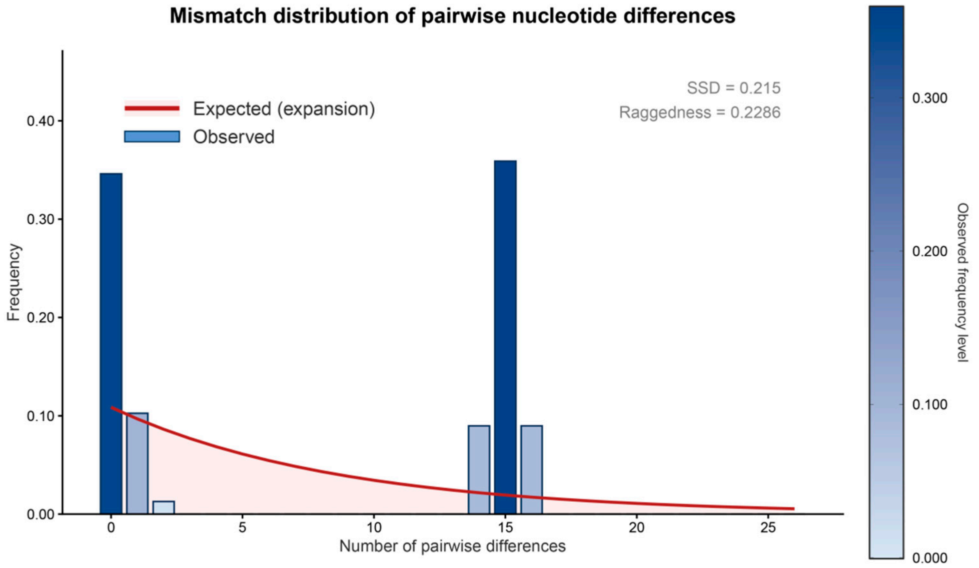

Figure S6. Mismatch distribution analysis of the Hainan gibbon (*Nomascus hainanus*) based on complete mitochondrial genome sequences. The observed distribution of pairwise nucleotide differences (blue bars) was compared with the expected distribution under a sudden expansion model (red curve). The sum of squared deviations (SSD) and raggedness index values are shown to evaluate the fit between observed and expected distributions.

Table S1: Primer sequences

Name	Direction	Sequence
ATP6-F	Forward	CACAAGTGAACGAAAATCTATTCGC
ATP6-R	Reverse	TTATGTGTTGTCATGCAGGTAGAG
ATP8-F	Forward	ATGCCCCAATTAAATACCACC
ATP8-R	Reverse	CTAGGATTGTGGGGGCAG
CON-E56-F	Forward	CTATTTCAGATGGGGGTCCC
CON-E56-R	Reverse	CAGTGTTTTGCTTTAGGTGGT
COX1-F	Forward	ACTAATGTTCGCCGACCG
COX1-G1-F	Forward	TCCACAAACCATAAAGATATTGGA
COX1-G1-R	Reverse	GAGGTTTCATGTTGATGATCGTA
COX1-R	Reverse	TCTAGGTTTTATATAGACGGGCTCT
COX2-F	Forward	ATGGCACATGCAACTCAAGT
COX2-R	Reverse	TTACAGGGTAAATACGGGTCCT
COX3-F	Forward	ATGACCCACCAATCACATGC
COX3-R	Reverse	AGGATCCTCATCAGTAGATGGAGAC
COX-B04-F	Forward	TCTGACTCCTCCCTCCCTC
COX-BO4-R	Reverse	GTGGAGTGTGGCGAGTCAG
CYTB-BO4-F	Forward	CTTTCACTTCATCCTACCCTTT
CYTB-BO4-R	Reverse	AATTTTAGCTTTGGGTGCTG

Name	Direction	Sequence
CYTB-F	Forward	ATGACTCCCCTACGCAAAAC
CYTB-R	Reverse	AGGGCAGGTCCATTTGAGTA
ND1-F	Forward	ATGCCCACAACCAACCTTCT
ND1-R	Reverse	TTCTTAGGTTTGGGGGGGAATG
ND2-BO4-F	Forward	ATGATAGCCATCCTCCCAT
ND2-BO4-R	Reverse	CCTTGGATGTTGTTGCAGAAATTA
ND2-F	Forward	ATCAACCCCCTAGCCCAACCTAT
ND2-R	Reverse	ATAGGGCAGGGGATGTGAGTGG
ND3-E55-F	Forward	CCACAACTCAACACCTACATAGA
ND3-E55-R	Reverse	GTTGGCTTGGCTGAGGAATA
ND3-F	Forward	GAGTAATGAACCTAACATTAGCCA
ND3-R	Reverse	ACTCGACCCAGTCTAGTC
ND4-BO4-F	Forward	TGACTAGCATGCATAATAGCC
ND4-BO4-R	Reverse	CGAATAGTTCTCCTAGGAGGTT
ND4-E55-R	Reverse	GTGGCGGTGAATGTTATGATTAG
ND4-E56-F	Forward	TGTCCAACGAATCCCTCCT
ND4-E56-R	Reverse	GTCTGTTTGTCGTAGACAGATG
ND4L-F	Forward	ATGCCCCTCATCTACATAAACA
ND4L-R	Reverse	TTAGCATTGGAGCAGGTTTAGG
ND4-R	Reverse	TATGTCTACAGGGAGTAGGGCC
ND5-E55-F	Forward	AACAAATCAACCCCGCTT
ND5-E55-R	Reverse	TTGAGATTGCTCGGGGTAA
ND5-G1-F	Forward	CGGCGATATCGGCTTTAT
ND5-G1-R	Reverse	GGGCACAAATTGCTGTAAATAGG
ND5-R	Reverse	GGTTATATAACTAGAAGGAGGGTTAGG
ND6-F	Forward	CCTATTACCCCGAGCAATCTC
ND6-R	Reverse	CCCATGCGAGAATGATGACATATA
con-F	Forward	TCCTACCTTAGACCAACCCAAC
con-R	Reverse	GGCTACCACATGAGAATACAGAGA
RRNL-F	Forward	GCCAACCCTAGCCCCAAACT
RRNL-G2-F	Forward	GTTCAAGCTCAACATCATCCAC
RRNL-G2-R	Reverse	GCATTGATAGGCTAAAGCTCCAT
RRNL-R	Reverse	AGGCTCTGTTCTTGAGCGAGTT
RRNS-E55-F	Forward	GGAATGAAAGAAATTCACTCCGC
RRNS-E55-R	Reverse	TCAATCGTATGACCGCGG
rrns-F	Forward	AATAGGTTTGGTCCTAGCCT
rrns-R	Reverse	GTTTATCCAAGTGCACCTTCC

Table S2: Comparative mitogenomic characteristics of representative Hylobatidae taxa

**Taxon**	**GenBank accession**	**Genome length (bp)**	**A+T (%)**	**Mean p-distance vs. *N. hainanus* (%)**
Nomascus hainanus	PZ236444–PZ236456	16,481	54.77	—
Nomascus concolor	PQ740954.1	16,468	55.08	5.137
Nomascus leucogenys	NC_021957.1	16,478	54.90	5.188
Nomascus siki	NC_014051.1	16,476	54.83	5.096
Nomascus gabriellae	NC_018753.1	16,478	54.89	5.287
Hylobates lar	NC_002082.1	16,472	54.55	9.954
Hoolock hoolock	NC_033885.1	16,495	55.59	9.552
Symphalangus syndactylus	NC_014047.1	16,516	56.48	9.177

All representative mitogenomes contained the canonical 13 protein-coding genes, 22 tRNAs, 2 rRNAs, and one control region in the conserved gibbon gene order. Uncorrected p-distances for external taxa are means across comparisons with all 13 N. hainanus mitogenomes; positions containing gaps in pairwise global alignments were excluded. Within N. hainanus, the mean p-distance between the two major maternal clades was 0.091% (range 0.085–0.097%; 14–16 substitutions across 16,481 bp). For population-genetic context, published whole-mitogenome nucleotide diversity values span 0.00071–0.01671 in Hylobates and 0.00253–0.00432 in sampled Nomascus taxa, including π = 0.00253 in N. concolor (Liu et al., 2025; reference 29 in the main manuscript).

Table S3: Annotation of the mitochondrial genome

Start	End	Length	Strand	Gene
1	70	70	+	trnF-TTC
71	1022	952	+	rrnS
1023	1091	69	+	trnV-GTA
1092	2648	1557	+	rrnL
2649	2723	75	+	trnL-TTA
2726	3681	956	+	ND1
3682	3750	69	+	trnI-ATC
3748	3819	72	-	trnQ-CAA
3820	3887	68	+	trnM-ATG
3888	4929	1042	+	ND2
4930	4998	69	+	trnW-TGA
5009	5077	69	-	trnA-GCA
5079	5152	74	-	trnN-AAC
5155	5184	30	+	rep_origin
5184	5249	66	-	trnC-TGC
5249	5314	66	-	trnY-TAC
5325	6866	1542	+	COX1

Start	End	Length	Strand	Gene
6867	6935	69	-	trnS-TCA
6939	7007	69	+	trnD-GAC
7008	7691	684	+	COX2
7719	7788	70	+	trnK-AAA
7790	7996	207	+	ATP8
7951	8631	681	+	ATP6
8631	9414	784	+	COX3
9415	9482	68	+	trnG-GGA
9483	9828	346	+	ND3
9829	9894	66	+	trnR-CGA
9895	10191	297	+	ND4L
10185	11559	1375	+	ND4
11560	11628	69	+	trnH-CAC
11629	11687	59	+	trnS-AGC
11688	11758	71	+	trnL-CTA
11765	13570	1806	+	ND5
13571	14095	525	-	ND6
14096	14164	69	-	trnE-GAA
14169	15309	1141	+	CYTB
15310	15375	66	+	trnT-ACA
15378	15445	68	-	trnP-CCA
15446	16481	1036	+	D-loop

Table S4: Codon usage bias

Amino_Acid	Codon	Mean_RSCU
Ala	GCC	1.933
Ala	GCA	1.062
Ala	GCU	0.874
Ala	GCG	0.131
Arg	CGA	1.694
Arg	CGC	1.441
Arg	CGU	0.577
Arg	CGG	0.288
Asn	AAC	1.425
Asn	AAU	0.575
Asp	GAC	1.538
Asp	GAU	0.462
Cys	UGC	1.333
Cys	UGU	0.667
Gln	CAA	1.568
Gln	CAG	0.432
Glu	GAA	1.348
Glu	GAG	0.652
Gly	GGC	1.756
Gly	GGA	1.244
Gly	GGG	0.533
Gly	GGU	0.467
His	CAC	1.313
His	CAU	0.687
Ile	AUC	1.16

Amino Acid	Codon	Mean RSCU
Ile	AUU	0.84
Leu1	CUA	2.031
Leu1	CUC	1.11
Leu1	CUG	0.438
Leu1	CUU	0.42
Leu2	UUA	1.404
Leu2	UUG	0.596
Lys	AAA	1.636
Lys	AAG	0.364
Met	AUA	1.444
Met	AUG	0.556
Phe	UUC	1.404
Phe	UUU	0.596
Pro	CCC	1.69
Pro	CCA	1.169
Pro	CCU	0.883
Pro	CCG	0.258
Ser1	AGC	1.5
Ser1	AGU	0.5
Ser2	UCA	1.555
Ser2	UCC	1.484
Ser2	UCU	0.707
Ser2	UCG	0.254
Thr	ACA	1.624
Thr	ACC	1.546
Thr	ACU	0.599
Thr	ACG	0.231
Trp	UGA	1.511
Trp	UGG	0.489
Tyr	UAC	1.174
Tyr	UAU	0.826
Val	GUC	1.363
Val	GUA	1.255
Val	GUG	0.706
Val	GUU	0.676
